# Case of a Bony Tumor Obstructing the Pylorus

**Published:** 1827-05

**Authors:** John Webster

**Affiliations:** Physician to the St. George's and St. James's Dispensary.


### BONY TUMOR IN THE STOMACH.

*Case of a Bony Tumor obstructing the Pylorus.* By John Webster, m.d. Physician to the St. George's and St. James's Dispensary.

It is remarked by pathologists, that bony concretions in the stomach or intestines are of exceedingly rare occurrence. Dr. Baillie has once or twice observed osseous matter formed upon the surface of the mucous membrane of the gut, but never in the stomach. Fungous tumors growing from narrow pedicles attached to that viscus, and so near to the pylorus as sometimes to obstruct even the passage of fluids into the duodenum, have occasionally been met with; but very few examples are recorded in which cartilaginous or bony substances have been found adhering to the stomach. As an instance of the latter, and of what is considered an unfrequent disease, the following case, terminating fatally from obstruction of the pylorus, is now communicated.

A gentleman, of rather a stout habit of body, and about sixty-two years of age, had for the most part enjoyed good health, with the exception of some common dyspeptic complaints, attended occasionally with costiveness, which, upon the supervention of a diarrhœa, were generally relieved. These attacks occurred at irregular intervals, but never assumed a severe character till last autumn; when, early one morning, after having the previous evening ate freely of damson-tart, he was seized with excruciating pain at the epigastrium, accompanied by slight sickness and an acceleration of pulse; the skin became warm, and there was thirst, great anxiety, with obstinately costive bowels.

These symptoms increasing in urgency, along with a sense of fulness and fluctuation at the epigastrium, Mr. NICHOLSON, of Davies-street, (who had now been sent for,) bled the patient from the arm, and gave repeated doses of purgative and saline medicines. He also directed a strong enema to be administered.

In the evening, I was requested to visit Mr. ——; when the symptoms were considerably increased, especially the pain, tension, and swelling at the epigastrium, which seemed to augment with the quantity of liquids swallowed. A large blister was applied to the stomach; Calomel and Colocynth, with Infusion of Senna and Epsom Salts, were prescribed, and another strong enema was repeated. Brandy and water was frequently given, as the patient began to sink; and it was intended to try Croton-oil, but no opportunity was afforded, for, after vomiting profusely, the patient died, about twenty-two hours from the commencement of the attack.

Next morning, Mr. Nicholson examined the body, in the presence of Mr. Barrow and myself. On opening the stomach, which was much distended with the fluids taken during the disease, a cartilaginous body, intermixed with numerous spiculæ of bone, nearly the size of a quart-bottle cork, was discovered firmly attached by one extremity to the coats of the stomach, close to the pylorus, into which the other projected like a stopper, thereby preventing any passage into the small intestines. The internal membrane of the stomach was slightly inflamed, as were the peritoneal and mucous coats, at several points of the duodenum and ileum. There were about three pounds of serum effused into the cavity of the abdomen.

If it had been possible accurately to ascertain during life that a mechanical obstruction, similar to the one now described, existed, recourse would naturally, in the first instance, have been had to an emetic, with a view to dislodge the tumor from its situation in the pylorus; but, as a condition like the present could not be anticipated, the treatment was therefore such as is usually employed in inflammatory affections of the bowels; the nature and extent of the remedies having been regulated according to the urgency of the symptoms. How these proved ineffectual, the post-mortem examination satisfactorily explains.

56, *Grosvenor-street; February*, 1827.

